# Notes on the nomenclature of *Artemisia* (I): *A.* subg. *Ponticae* (Asteraceae)

**DOI:** 10.3897/phytokeys.281.210520

**Published:** 2026-10-29

**Authors:** Yifan Liu, Alexey P. Seregin, Guohao Niu, Dahai Zhu, Artem Leostrin, Denis A. Krivenko, Bohan Jiao, Tiangang Gao

**Affiliations:** 1 State Key Laboratory of Plant Diversity and Specialty Crops, Institute of Botany, Chinese Academy of Sciences, Beijing 100093, China State Key Laboratory of Plant Diversity and Specialty Crops, Institute of Botany, Chinese Academy of Sciences Beijing China; 2 China National Botanical Garden, Beijing 100093, China China National Botanical Garden Beijing China; 3 University of Chinese Academy of Sciences, Beijing 100049, China University of Chinese Academy of Sciences Beijing China; 4 Faculty of Biology, M. V. Lomonosov Moscow State University, Moscow 119991, Russia Faculty of Biology, M. V. Lomonosov Moscow State University Moscow Russia; 5 Dujiangyan Station of Giant Panda National Park, Chengdu 611830, China Dujiangyan Station of Giant Panda National Park Chengdu China; 6 Komarov Botanical Institute, Russian Academy of Sciences, St. Petersburg 197022, Russia Komarov Botanical Institute, Russian Academy of Sciences St. Petersburg Russia; 7 Siberian Institute of Plant Physiology & Biochemistry, Siberian Branch of the Russian Academy of Sciences, Irkutsk 664033, Russia Siberian Institute of Plant Physiology & Biochemistry, Siberian Branch of the Russian Academy of Sciences Irkutsk Russia

**Keywords:** *Artemisia* subg. *Ponticae*, Asteraceae, lectotype, nomenclature

## Abstract

Lectotypes are designated for five plant names in *Artemisia* subg. *Ponticae*: *A. afra* Jacq. ex Willd., *A. brachyloba* Franch., *A. mesatlantica* Maire, *A. persica* Boiss., *A. subspinescens* Boiss. An error in the holotype citation of *A. aksaiensis* Y.R.Ling is corrected. These actions stabilize the application of these species names and provide a basis for further revision of the subgenus.

## Introduction

*Artemisia* L. (Asteraceae), comprising approximately 500 species mainly distributed in the Northern Hemisphere, is a big genus of significant medicinal and ecological importance (Jiao et al. 2023, 2025). The main diagnostic characters of the genus are as follows: annual, biennial, or perennial herbs, subshrubs, or shrubs, often aromatic; leaves entire to pinnatisect or pectinately pinnatisect; capitula small, heterogamous-disciform or homogamous-discoid; receptacles glabrous or pubescent; disk florets bisexual and fertile or functionally staminate; pollen of the *Artemisia* type, spineless or shortly spined; achenes without ribs (Bremer 1993; Ling et al. 2011; Jiao et al. 2023). Recent phylogenomic and morphological studies recognize eight subgenera—*Artemisia*, *Absinthium*, *Dracunculus*, *Pectinatae*, *Pacifica*, *Ponticae*, *Seriphidium*, and *Tridentatae*—comprising 24 sections (Jiao et al. 2023, 2025, 2026). Among these, subg. *Ponticae* is mainly diagnosed by a subshrub or shrub habit, strongly aromatic foliage, 2-pinnatisect or 2-pectinately pinnatisect leaves with linear, filiform, or lanceolate lobes, paniculate synflorescence, heterogamous-disciform capitula, glabrous receptacles, and achenes without ribs and without a crown; the subgenus contains 23 species, mainly distributed in temperate Eurasia, with a few species in Africa (Jiao et al. 2025).

Although this classification framework is now available, the nomenclature of *Artemisia* remains challenging because of its large number of species and a complex naming history spanning more than two centuries (Jiao et al. 2023; Boiko et al. 2025). Many species were described in the 18^th^ and 19^th^ centuries, long before the introduction of the type concept; as a result, numerous names remain untypified or may contain errors.

We are now revising the taxonomy of *Artemisia* subg. *Ponticae* B.H.Jiao & T.G.Gao in Jiao et al. (2023). During this process, it was necessary to designate lectotypes for the following five species names: *A. afra* Jacq. ex Willd., *A. brachyloba* Franch., *A. mesatlantica* Maire, *A. persica* Boiss., and *A. subspinescens* Boiss., as no holotype was indicated in their protologues, and no effective lectotypification had subsequently been published. In addition, an error in the holotype citation of *A. aksaiensis* Y.R.Ling needs to be corrected. This is the first part of the work on the nomenclature of the genus *Artemisia*. Follow-up investigations on other subgenera are currently in progress and will be submitted in due course.

## Material and methods

Herbarium specimens, especially types and original material, deposited at A, AL, B, BM, FI, FR, G, GOET, H, HAL, HBG, K, LE, M, MO, MPU, MW, P, PE, RAB, S, US, W, and WAG were examined physically or as digital images. Digital images were obtained from the online herbarium databases of B (http://herbarium.bgbm.org/), P (https://science.mnhn.fr/), G (https://collections.geneve.ch/jardinbotaniquegeneve/herbier/), and other herbaria. Images of the newly designated lectotypes are provided directly in this paper. Bibliographical citations in the original publications and in nomenclatural databases such as IPNI (International Plant Names Index; http://ipni.org/) and Tropicos (http://www.tropicos.org/) were also checked.

In lectotypifying plant names, we followed the International Code of Nomenclature for algae, fungi, and plants (Madrid Code) (Turland et al. 2025). When a protologue cited more than one gathering without indicating a holotype, the cited specimens are syntypes under Art. 9.6, and the lectotype is selected from among them. If no specimen was explicitly cited in the protologue, the lectotype was chosen from the original material (Arts. 9.3 and 9.4), using the history of collections to trace the specimen that best matches the protologue. An error in a holotype citation was corrected following Art. 9.2.

All type specimens cited in this work were examined by the authors, either physically or as digital images.

## Nomenclatural treatment

### 1. Artemisia afra

Taxon classification

Plantae

AsteralesAsteraceae

Jacq. ex Willd., Sp. Pl., ed. 4, 3(3): 1820. 1803.

19E093A0-FB08-5D27-BD84-8A81898DD817

#### Type.

**Figure 1.**
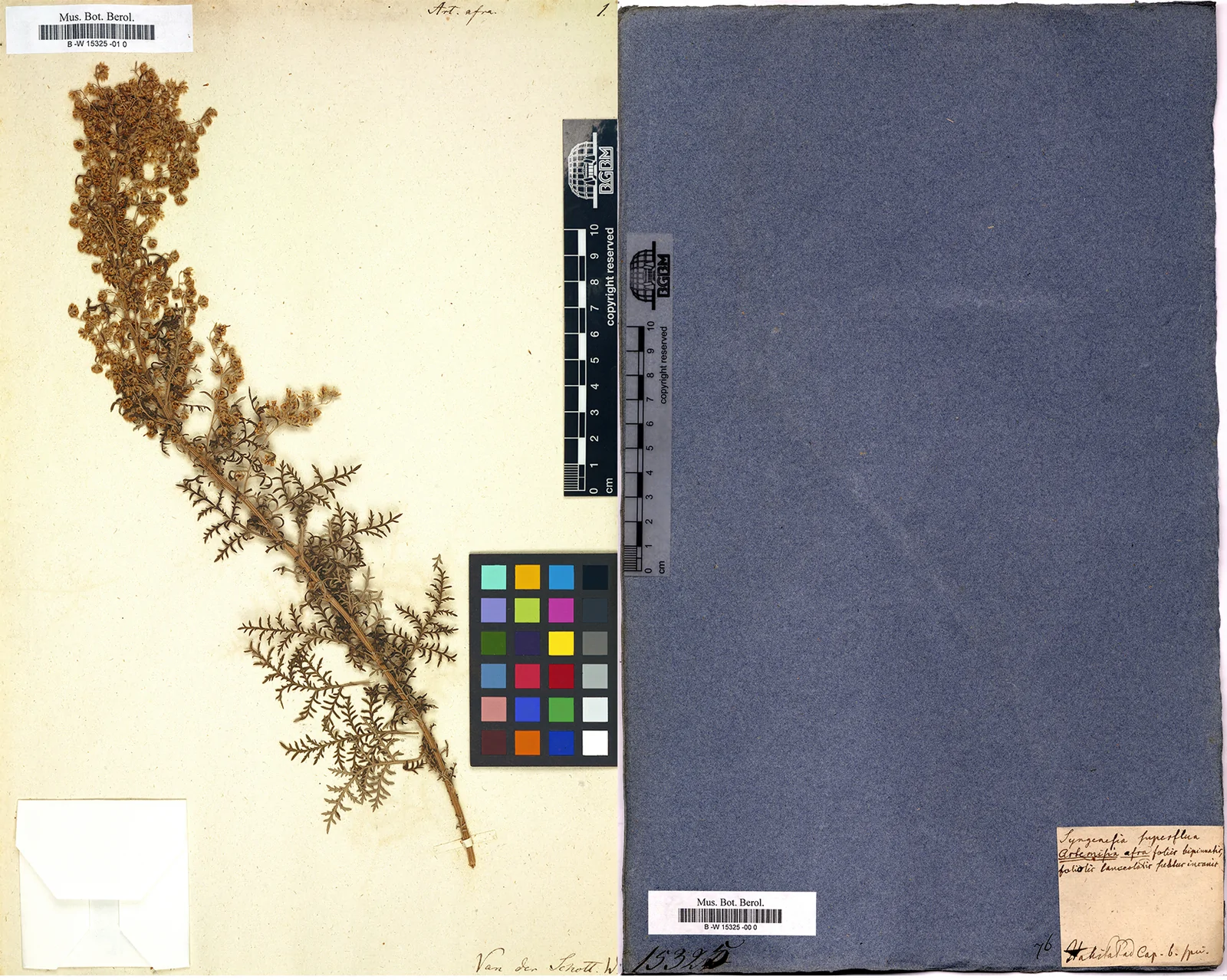
Lectotype of *Artemisia afra* Jacq. ex Willd. (B-W-15325-01 0). Digital image from the Botanic Garden and Botanical Museum Berlin (B). Available at: http://herbarium.bgbm.org/object/BW15325-010 (accessed 24 September 2026).

South Africa • Cap. b. sp. [Caput bonae spei], s.d., *Richard van der Schot s.n*. (lectotype, designated here, B [B-W-15325-01 0!] (Fig. 1); isolectotype: HBG [HBG0504025!]).

#### Note.

The name *Artemisia afra* was first proposed by N.J. von Jacquin and subsequently validly published by C.L. Willdenow with a formal description. The protologue provides a morphological description and a German vernacular name and records the locality as “Cap. b. spei?” (an abbreviation of “Caput Bonae Spei,” the Cape of Good Hope, South Africa), the question mark indicating Willdenow’s uncertainty about the exact locality. It is also annotated “(v. v.)” (*vidi vivam*), indicating that the author had seen living plants. The protologue did not cite any specific specimen or collector number, and since the type method was not yet established at the time, no type was indicated. Therefore, the original material comprises the specimens in Willdenow’s herbarium that can be associated with this description, as well as any duplicates later separated from this herbarium.

The Willdenow herbarium, maintained as an independent collection, was acquired in its entirety by the Berlin Herbarium (B) in 1818 and is among the holdings that survived the 1943 fire (Hiepko 1972; Stafleu 1972). Specimen B-W-15325-01 0 in this collection exhibits characters of habit, leaf morphology, indumentum, inflorescence, and involucre that all agree with the protologue (Willdenow 1803). The annotation “Van der Schott. W.” on the front of the specimen confirms that it came from Richard van der Schot, with the “W.” being a mark added by Schlechtendal when curating the collection to indicate Willdenow’s ownership (Hiepko 1972). Van der Schot worked at the Imperial Gardens of Schönbrunn alongside Jacquin (D’Arcy 1970); the living plants observed by Willdenow and mentioned in the protologue very likely originated from that garden, and the material was seemingly provided by van der Schot. Although the protologue gives the locality as “Cap. b. spei?”, the specimen therefore represents cultivated material derived from South African stock. On the back of the specimen folder is a label consistent with the typical format of Willdenow’s herbarium; the diagnosis written on the label agrees with the protologue (Willdenow 1803). This specimen is part of the original material used by Willdenow to publish the species and therefore constitutes original material as defined by the Madrid Code (Turland et al. 2025).

Only two original specimens could be located: B-W-15325-01 0 (B) and HBG0504025 (HBG). Among the original material examined, B-W-15325-01 0 is the most suitable choice for the lectotype because it bears a well-developed and complete plant with fully developed inflorescences and numerous capitula and because its original label and annotations directly link it to Willdenow’s protologue (as detailed above). Taxonomically, it fully represents the diagnostic characters of the species as described in the protologue and is therefore the most appropriate element to fix the application of the name. Specimen HBG0504025, housed at the Hamburg Herbarium (HBG), originally belonged to the Willdenow collection and was later separated by Schlechtendal into his personal collection. This plant is smaller and bears fewer capitula, and the specimen lacks the original Willdenow herbarium label and mark and is therefore less informative for fixing the application of the name. Accordingly, B-W-15325-01 0 is here designated as the lectotype of *Artemisia afra*. The HBG specimen is a duplicate of the lectotype and is treated here as an isolectotype.

#### Taxonomic acceptance.

*Artemisia afra* is currently accepted as a distinct species (POWO 2026).

### 2. Artemisia brachyloba

Taxon classification

Plantae

AsteralesAsteraceae

Franch., Nouv. Arch. Mus. Hist. Nat., sér. 2, 6: 51. 1883.

6A5CE453-D4E1-5DE2-8173-114D123B4273

#### Type.

**Figure 2.**
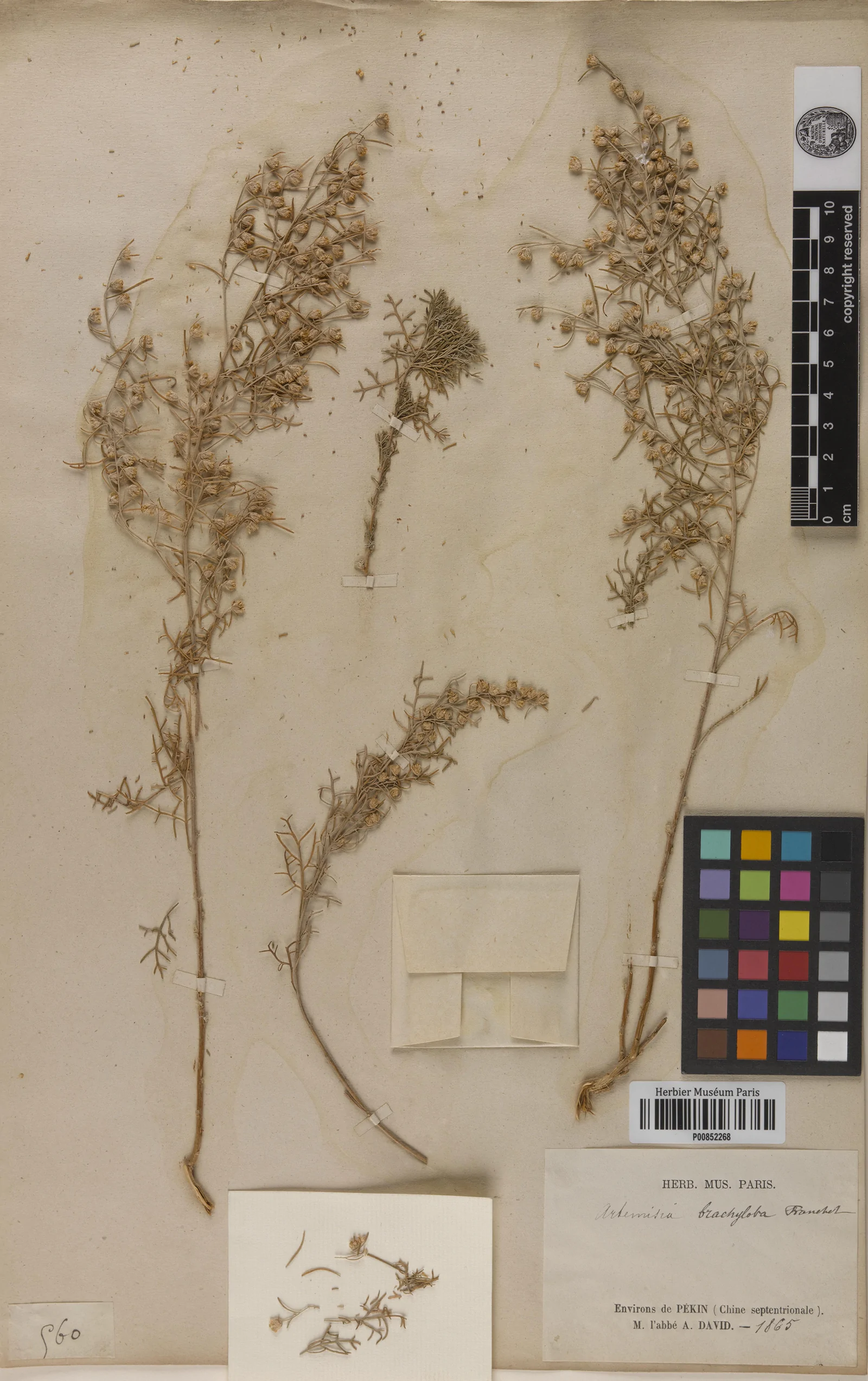
Lectotype of *Artemisia brachyloba* Franch. (P00852268). Digital image from the Muséum national d’Histoire naturelle (P). Available at: https://science.mnhn.fr/institution/mnhn/collection/p/item/p00852268 (accessed 24 September 2026).

China • Environs de Pékin (Chine septentrionale), 1865, *David 560* (lectotype, designated here, P [P00852268!]) (Fig. 2).

#### Note.

In the protologue, Franchet (1883: 51) cited two gatherings, “Chine: environs de Pékin (n° 560)” and “Mongolie: Si-wan, septembre 1862,” but no holotype was indicated. Therefore, both gatherings are syntypes under Art. 9.6. The original material of *A. brachyloba* thus comprises the specimens corresponding to these two gatherings. The specimens are now deposited at P.

Both gatherings were collected by Armand David (Abbé A. David), a French missionary and naturalist who collected extensively in China. The first corresponds to the surroundings of present-day Beijing, China. The second, “Si-wan,” corresponds to Xiwanzi (西湾子), now in Chongli District, Zhangjiakou, Hebei Province, China, a historic Catholic mission station located just north of the Great Wall and approximately 30 miles northeast of Kalgan (present-day Zhangjiakou) (David 1867).

Specimen P00852268 bears the collector’s number “560,” matching exactly the first gathering cited. Its original label reads: “Environs de PÉKIN (Chine septentrionale). M. l’abbé A. DAVID. — 1865.” The locality and collector’s number on this label correspond directly to Franchet’s citation; the label further records that it was collected by David in 1865. Morphologically, the plant has bipinnatisect leaves with the abaxial surface densely whitish-canescent, hemispherical and subnutant capitula, lanate outer involucral bracts, and a lax paniculate-racemose inflorescence, all of which agree in detail with the protologue (Franchet 1883). Specimen P00852269 corresponds to the second gathering cited, but its label lacks an explicit collector’s number.

Among the original material, P00852268 is selected as the lectotype because it is the most complete and informative element: it bears the collector’s number that exactly matches the first gathering cited in the protologue, its label information is complete and unambiguous, and its morphological characters fully agree with the original description. In contrast, P00852269 lacks an explicit collector’s number. Accordingly, P00852268 is here designated as the lectotype of *A. brachyloba*. Following this designation, P00852269 is treated as a remaining syntype.

#### Taxonomic acceptance.

*Artemisia brachyloba* is currently accepted as a distinct species (POWO 2026).

### 3. Artemisia mesatlantica

Taxon classification

Plantae

AsteralesAsteraceae

Maire, Bull. Soc. Sci. Nat. Maroc 8: 138. 1928.

7062FECC-F8DD-5AEA-8C4B-3211DDD79BB1

#### Type.

**Figure 3.**
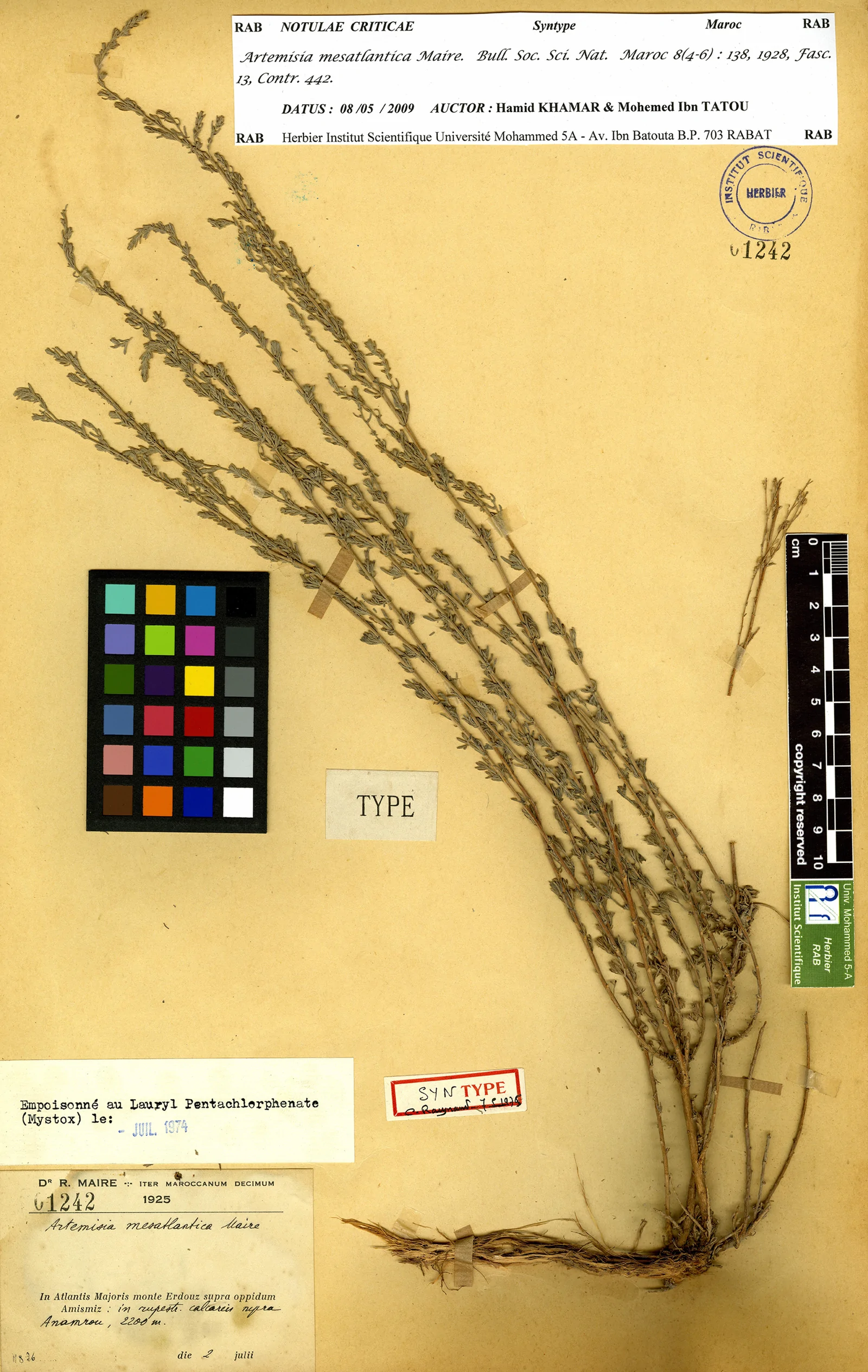
Lectotype of *Artemisia mesatlantica* Maire (RAB001242). Digital image from the Institut Scientifique, Rabat (RAB). Available at: https://science.mnhn.fr/institution/rabat/collection/rab/item/rab001242 (accessed 24 September 2026).

Morocco • In Atlantis Majoris monte Erdouz supra oppidum Amismiz, in rupibus calcareis supra Anamrou, 2,200 m, 2 Jul 1925, *R. Maire 01242* (lectotype, designated here, RAB [RAB001242!] (Fig. 3); isolectotype: MPU [MPU038183!]).

#### Note.

In the protologue, Maire (1928: 138) cited four expeditions: (1) Humbert & Maire 1923, from the plateau near Tinhadit and the mountains Hayan and Benij in the Middle Atlas; (2) Humbert & Maire 1925, from Mount Erdouz in the High Atlas; (3) Litardière & Maire 1926, from Mount Anremer in the High Atlas; and (4) Humbert’s collection from the eastern mountains such as Ayachi. The protologue stated that the types were deposited at the Herbarium of the University of Algiers (AL) and the Herbarium of the Imperial Scientific Institute in Rabat (RAB), but no holotype was indicated. Therefore, all cited specimens constitute syntypes under Art. 9.6.

Maire’s specimens were transferred from AL to MPU in the 1960s, with some duplicates in RAB and P, allowing the examination of the material originally deposited at AL. We searched the major herbaria likely to hold Maire’s specimens (including MPU, H, RAB, P, K, etc.) and examined the digitized syntypes. A total of 11 specimens of original material were located, belonging to seven gatherings that cover all four expeditions cited in the protologue:

Gathering 1 (first expedition): Middle Atlas, near Aghbalou Larbi, clay soil, ca. 2,000 m, 10 Aug 1924, MPU001954 (MPU), H1649773 (H), P03307507 (P, originally from AL).

Gathering 2 (first expedition): Middle Atlas, Tizi-n-Taghzeft, limestone, 1,900–2,100 m, 10 Aug 1924, MPU001955 (MPU), P03307508 (P, originally from AL).

Gathering 3 (first expedition): Middle Atlas, basaltic plateau between Tinhadit and Ari Benij, Sep 1924, MPU038187 (MPU, originally from AL).

Gathering 4 (second expedition): High Atlas, Mount Erdouz, above Amismiz to Anamrou, limestone, 2,200 m, 2 Jul 1925, RAB001242 (RAB), MPU038183 (MPU, originally from AL).

Gathering 5 (second expedition): High Atlas, Mount Erdouz, near Amismiz, limestone, 2,200 m, 2 Jul 1925, P03307506 (P, originally from AL).

Gathering 6 (third expedition): High Atlas, Mount Anremer, limestone, 2,200–3,000 m, 27 Jul 1926, MPU001953 (MPU).

Gathering 7 (fourth expedition): Eastern Morocco, desert mountains, 2,500–3,000 m, 1926, P02698574 (P).

Among the listed syntypes, RAB001242 has a complete woody rhizome, clearly visible leaf morphology, and well-preserved indumentum; its diagnostic characters are the best preserved among the syntypes. Furthermore, RAB is one of the two institutions explicitly designated in the protologue for the deposition of types, and selecting a specimen originally deposited there better reflects the author’s original intent. Taxonomically, it fully represents the diagnostic characters of the species as described in the protologue. Therefore, RAB001242 is designated here as the lectotype of *Artemisia mesatlantica*. Specimen MPU038183 matches the lectotype precisely in collector, collection number, date, and locality and is a duplicate of the same gathering; it is consequently an isolectotype. The remaining nine specimens belong to different gatherings and are treated as remaining syntypes.

#### Taxonomic acceptance.

*Artemisia mesatlantica* is currently accepted as a distinct species (POWO 2026).

### 4. Artemisia persica

Taxon classification

Plantae

AsteralesAsteraceae

Boiss. in Hohenacker, Pl. Pers. austr.: no. 630. 1845.

87D26620-F527-5657-A93B-D4B769CEDA95

#### Type.

**Figure 4.**
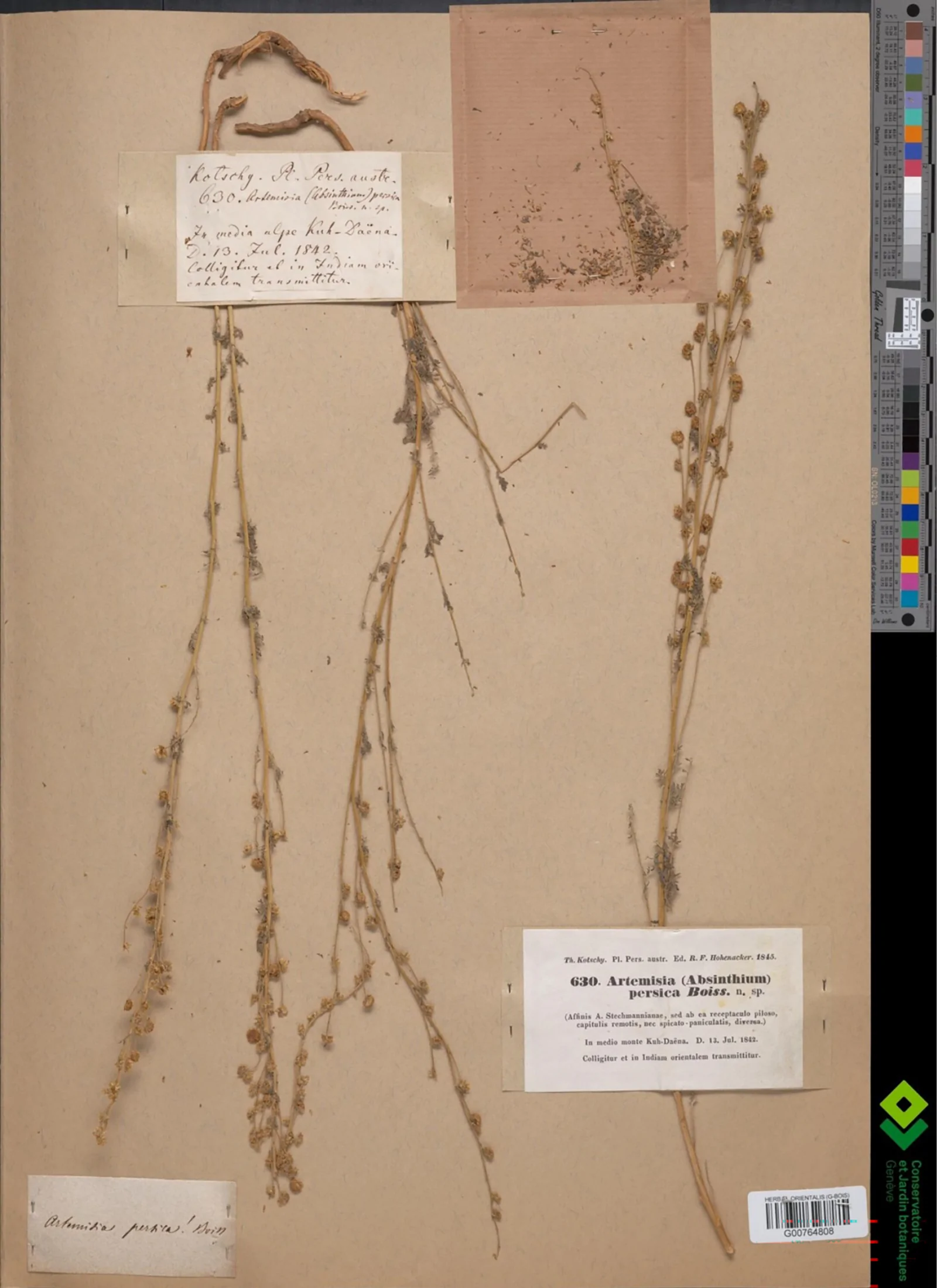
Lectotype of *Artemisia persica* Boiss. (G00764808). Digital image from the Conservatoire et Jardin botaniques de la Ville de Genève (G), Boissier’s Flora Orientalis collection (G-BOIS), via the Catalogue de l’herbier de Genève (CHG). Available at: https://collections.geneve.ch/jardinbotaniquegeneve/herbier/adetail.php?id=544872 (accessed 24 September 2026).

Iran • In media alpe Kuh-Daëna, 13 Jul 1842, *Kotschy* in Hohenacker, Pl. Pers. austr. no. 630 (lectotype, designated here, G [G00764808!] (Fig. 4); isolectotypes: A [A00002808!], C [C10024705!, C10024706!], CAS [BOT-BC:1574153], E [E00574754!], FI [FI006525!], FR [FR0030802!], GOET [GOET001059!], H [H1092660!], HAL [HAL0111436!], JE [JE00008339!], K [K002718131!], L [L.3018755!], M [M0030258!, M0097247!], MO [MO-797676!, MO-2583784!], O [O-V-2017223!], P [P00711973!, P00711976!, P00711982!, P00711983!], S [S-G-657!], US [US00324288!], WAG [WAG0004199!, WAG0004200!]).

#### Note.

*Artemisia persica* was validly published on the printed label of Kotschy’s specimen 238 630. Between 1841 and 1843, Kotschy carried out extensive plant collecting in Iran (Lack 2020); the material was subsequently organized and distributed commercially by the Esslingen plant dealer R. F. Hohenacker, with the species identifications on the labels provided by Boissier (Hohenacker 1845; Wörz 2016). This set of specimens was intended for commercial sale rather than being a standard exsiccata serving taxonomic or educational purposes, and it falls into the category of gray literature in a taxonomic sense (Sennikov and Belyaeva 2025).

The printed label bears a short diagnosis of a new species, satisfying the requirements for valid publication under the Madrid Code (Turland et al. 2025). The label was distributed together with the specimens in 1845, predating Boissier’s later inclusion of the species in his monograph of new species from the Orient (Boissier 1846), and therefore, it was effectively published in 1845 under Arts. 6 and 30.8. The protologue itself is the printed label, which cites the gathering as “In medio monte Kuh-Daëna. D. 13. Jul. 1842”—a locality corresponding to the central part of Kuh-e Dena (Kuh-e Dinar), a high massif in the central Zagros Mountains of present-day Fars Province, Iran—and provides a short diagnosis. No holotype was indicated; therefore, all specimens bearing this printed label constitute original material.

Because Kotschy no. 630 was commercially distributed, its parts are deposited in several herbaria, including A, C, CAS, E, FI, FR, GOET, H, HAL, JE, K, L, M, MO, O, P, S, US, and WAG. All these specimens are duplicates of the same gathering and therefore constitute original material under Art. 9.6. Boissier’s personal herbarium is preserved in the collection “Herbarium – Boissier’s Flora Orientalis (CJBG-G-BOIS)” at the Conservatoire et Jardin botaniques de Genève (G). Within this collection, the only original material of *A. persica* is G00764808, which bears both the printed label that constitutes the protologue and a handwritten label. The printed label reads: “Affinis A. Stechmannianae, sed ab ea receptaculo piloso, capitulis remotis, nec spicato-paniculatis, diversa. In medio monte Kuh-Daëna. D. 13. Jul. 1842.” The handwritten label records the same collection information. Morphologically, the plant on the sheet has leaves that are 2–3-pinnatisect, and the capitula are remote, not arranged in a spiciform panicle, which fully agrees with the diagnostic description on the label. The specimen is well preserved.

Among the available original material, G00764808 is selected as the lectotype because it is the specimen from Boissier’s personal herbarium, bears both the printed label that constitutes the protologue and a handwritten label, and has morphological characters that fully agree with the diagnostic description. It is the most complete and authoritative original element, directly linked to the author’s own collection and intent. The other duplicates are treated as isolectotypes. Accordingly, specimen G00764808 is designated here as the lectotype of *Artemisia persica*.

#### Taxonomic acceptance.

*Artemisia persica* is currently accepted as a distinct species (POWO 2026).

### 5. Artemisia subspinescens

Taxon classification

Plantae

AsteralesAsteraceae

Boiss., Diagn. Pl. Orient., ser. 1, 6: 91. 1846.

252DDA14-9F10-5552-8A09-63BC12C1B630

≡ Artemisia persica var. subspinescens (Boiss.) Boiss., Fl. Orient. 3: 374. 1875.

#### Type.

**Figure 5.**
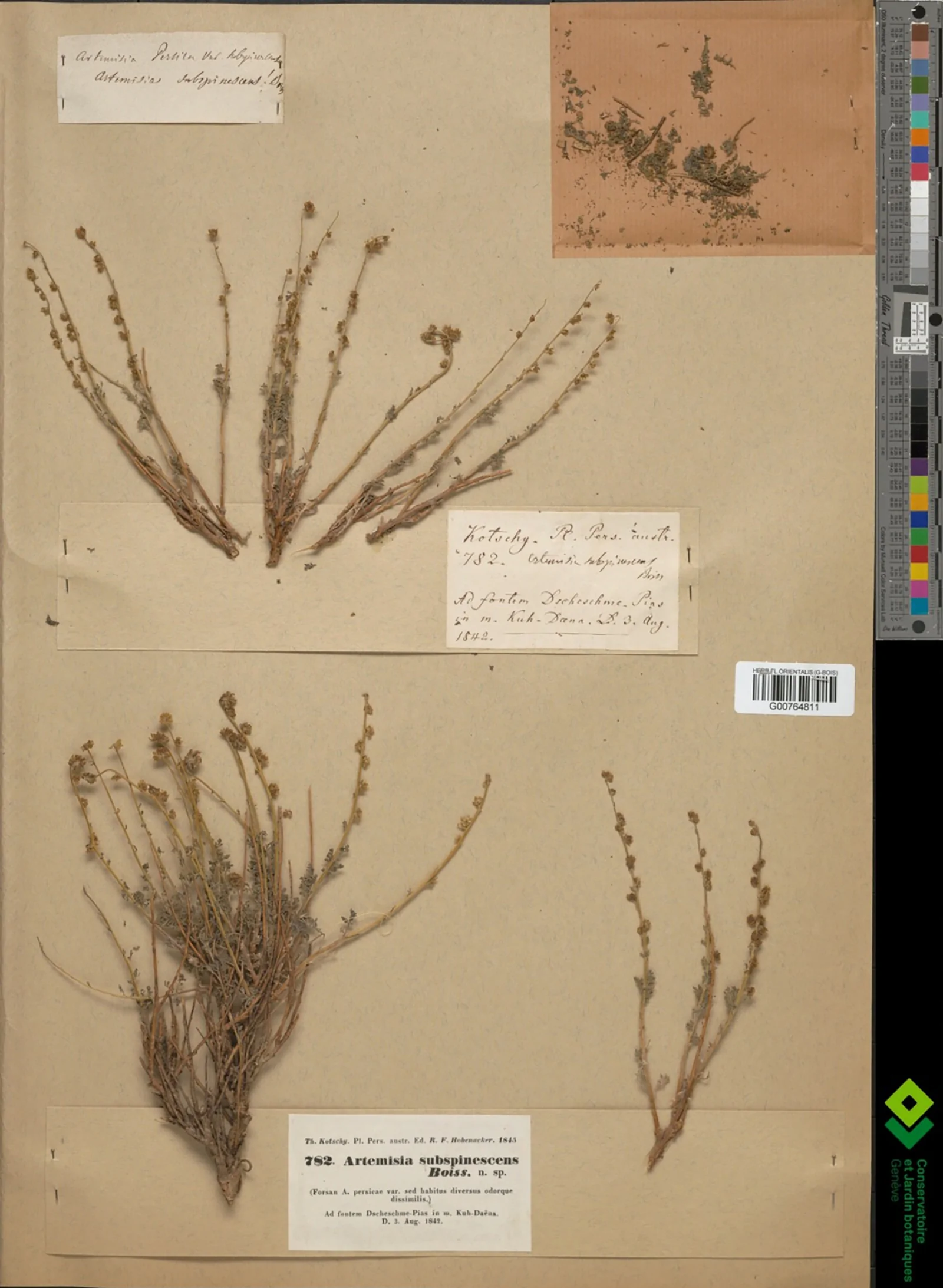
Lectotype of *Artemisia subspinescens* Boiss. (G00764811). Digital image from the Conservatoire et Jardin botaniques de la Ville de Genève (G), Boissier’s Flora Orientalis collection (G-BOIS), via the Catalogue de l’herbier de Genève (CHG). Available at: https://collections.geneve.ch/jardinbotaniquegeneve/herbier/adetail.php?id=544876 (accessed 24 September 2026).

Iran • Ad fontem Dscheschme-Pias in m. Kuh-Daëna, 3 Aug 1842, *Kotschy* in Hohenacker, Pl. Pers. austr. no. 782 (lectotype, designated here, G [G00764811!] (Fig. 5); isolectotypes: C [C10024707!, C10024708!], GH [GH00002813!, GH00002814!], GOET [GOET001058!], K [K000891958!, K002718130!], L [L.3018754!], M [M-0030257!], MO [MO2583781!], O [O:V:2017220!], P [P00711977!, P00711978!], WAG [WAG.1485090!], WIS [v0256539WIS!], WU [WU0172562!]).

#### Note.

*Artemisia subspinescens* was validly published by Boissier (1846: 91) in Diagn. Pl. Orient., where a full diagnosis and morphological description were provided and the gathering Kotschy no. 782 was cited. Although the same specimen had been distributed commercially by Hohenacker in 1845, its printed label bears only the tentative remark “Forsan *A. persicae* var. sed habitus diversus odorque dissimilis,” which does not constitute an effective diagnosis because no particular character states were mentioned. The valid publication therefore dates from Boissier’s 1846 work. No holotype was indicated; therefore, all specimens belonging to Kotschy no. 782 constitute original material.

Because Kotschy no. 782 was commercially distributed, its parts are deposited in several herbaria, including C, GH, GOET, K, L, M, MO, O, P, WAG, WIS, and WU. All these specimens are duplicates of the same gathering and therefore constitute original material under Art. 9.6. Boissier’s personal herbarium is preserved in the collection “Herbarium – Boissier’s Flora Orientalis (CJBG-G-BOIS)” at the Conservatoire et Jardin botaniques de Genève (G). Two original syntypes from Kotschy’s distribution no. 782 are held in this collection. The first, G00764811 (no. 782), bears both the printed label and a handwritten label; the latter reads: “Kotschy. Pl. Pers. austr. 782. *Artemisia subspinescens* Boiss. Ad fontem Dscheschme-Pias in m. Kuh-Daëna. D. 3. Aug. 1842.” It is clearly annotated “n. sp.,” indicating its status as a new species. Morphologically, this plant is a low, much-branched subshrub with canescent, shortly petiolate, bipinnatisect leaves and secund capitula borne on very short pedicels, all agreeing with the detailed description in the protologue (Boissier 1846). According to Edmondson and Lack (2006), Kotschy no. 782 was collected at Cheshmeh Piaz on Kuh-e Dena (30°55'N, 51°30'E), in present-day Kohgiluyeh and Boyer-Ahmad Province, Iran, in August 1842; this clarifies the locality cited in the protologue as “Ad fontem Dscheschme-Pias in m. Kuh-Daëna.”

The second syntype, G00764810 (no. 782b), bears a distribution label from Hohenacker but without a pre-printed species name, a type of label used for residual gatherings or incomplete collections. It was identified to the species by Boissier, but with a note that it was untypical (“var.”). Moreover, it was collected on a different date and is therefore a different syntype gathering; because of its deviating morphology and irregular distribution, it is not preferable for typification. This specimen is also fragmentary and poorly developed, making it less suitable for typification.

Among the original material, G00764811 is selected as the lectotype because it is better preserved, explicitly labeled as a new species, and in full agreement with the protologue. Taxonomically, it fully represents the diagnostic characters of the species as described by Boissier (1846). In contrast, G00764810 is a different gathering, has deviating morphology, is fragmentary, and is annotated as a variety, and is therefore less suitable for fixing the application of the name. Accordingly, G00764811 is designated here as the lectotype of *Artemisia subspinescens*. Specimen G00764810 remains a syntype, and the duplicates listed above are treated as isolectotypes.

#### Taxonomic acceptance.

*Artemisia subspinescens* is currently treated as a synonym of *A. persica* in POWO (2026).

##### Correction of the holotype citation of *Artemisia aksaiensis* Y.R.Ling

### Artemisia aksaiensis

Taxon classification

Plantae

AsteralesAsteraceae

Y.R.Ling in Bull. Bot. Res., Harbin 5(2): 3. 1985.

0AE4AEE8-2F32-5BA1-BDC7-8C6E22982E5B

#### Type.

**Figure 6.**
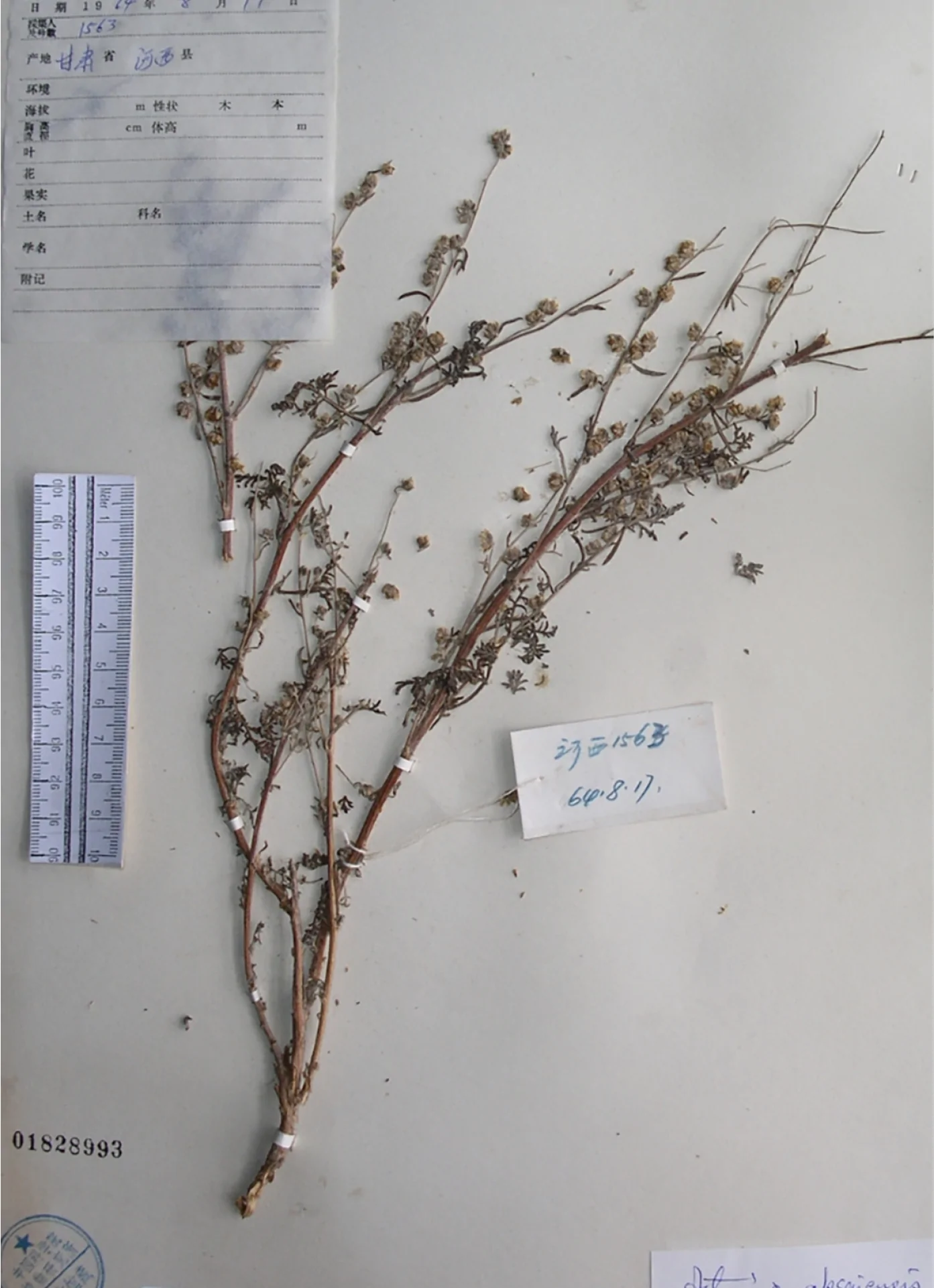
Holotype of *Artemisia aksaiensis* Y.R.Ling (PE00420308). Digital image from the Herbarium of the Institute of Botany, Chinese Academy of Sciences (PE), via the Chinese Virtual Herbarium (CVH). Available at: https://www.cvh.ac.cn/spms/detail.php?id=f40e429f (accessed 24 September 2026).

China • Gansu, Aksai, G.L.Chang 1563 (holotype, PE, Fig. 6; isotype: LZU).

#### Typification note.

In the protologue, Ling (1985: 3) cited the type as “G.L.Chang 1562, PE.” However, examination of the specimen bearing the number 1562 at PE reveals that it belongs not to *A. aksaiensis* but to *Stipa tianschanica*. A second specimen at PE, numbered 1563, was collected at the same locality (Aksai, Gansu Province), on the same date (17 August 1963), by the Hexi Expedition Team (河西队), of which G.L.Chang (张国梁) was a member. This specimen agrees completely with the protologue in its morphological characters and bears an identification label by Y.R.Ling. The collector, locality, and date are identical for both specimens, which differ only in the final digit of the collection number. It is therefore evident that “1562” in the protologue is a typographical error for “1563.” Under Art. 9.2 of the Madrid Code (Turland et al. 2025), such a typographical error does not invalidate the type designation; the intended holotype is specimen G.L.Chang 1563 (PE). The duplicate of this gathering is treated as an isotype of *A. aksaiensis*.

## Supplementary Material

XML Treatment for Artemisia afra

XML Treatment for Artemisia brachyloba

XML Treatment for Artemisia mesatlantica

XML Treatment for Artemisia persica

XML Treatment for Artemisia subspinescens

XML Treatment for Artemisia aksaiensis

## Additional information

### Artificial Intelligence (AI) use

The authors accept full responsibility for the content of the manuscript, including the disclosure of any use of AI.

No AI tools were used in the preparation of this manuscript.

### Author ORCIDs

Y. Liu https://orcid.org/0009-0008-1205-3560

A.P. Seregin https://orcid.org/0000-0002-1824-7453

G. Niu https://orcid.org/0009-0007-1436-9326

D. Zhu https://orcid.org/0009-0007-7812-5590

A. Leostrin https://orcid.org/0000-0002-9269-7954

D.A. Krivenko https://orcid.org/0000-0003-2658-1723

B. Jiao https://orcid.org/0000-0001-7621-3676

T. Gao https://orcid.org/0000-0001-8702-9647

### Data availability

All of the data that support the findings of this study are available in the main text.
